# Obesity Among HIV-Infected Adults Receiving Medical Care in the United States: Data From the Cross-Sectional Medical Monitoring Project and National Health and Nutrition Examination Survey

**DOI:** 10.1097/MD.0000000000001081

**Published:** 2015-07-13

**Authors:** Angela M. Thompson-Paul, Stanley C. Wei, Christine L. Mattson, McKaylee Robertson, Alfonso C. Hernandez-Romieu, Tanvir K. Bell, Jacek Skarbinski

**Affiliations:** From Division of HIV/AIDS Prevention (AMTP, SCW, CLM, MKR, ACHR, JS); Epidemic Intelligence Service, Centers for Disease Control and Prevention, Atlanta, Georgia (AMTP); United States Public Health Service, Rockville, Maryland (AMTP, SCW); Oak Ridge Institute for Science and Education (MKR); Department of Epidemiology, Rollins School of Public Health, Emory University, Atlanta, Georgia (ACHR); and Department of Internal Medicine, Division of Infectious Diseases, University of Texas Medical School at Houston, Houston, Texas, USA (TKB).

## Abstract

Our objective was to compare obesity prevalence among human immunodeficiency virus (HIV)-infected adults receiving care and the U.S. general population and identify obesity correlates among HIV-infected men and women.

Cross-sectional data was collected in 2009 to 2010 from 2 nationally representative surveys: Medical Monitoring Project (MMP) and National Health and Nutrition Examination Survey (NHANES).

Weighted prevalence estimates of obesity, defined as body mass index ≥30.0 kg/m^2^, were compared using prevalence ratios (PR, 95% confidence interval [CI]). Correlates of obesity in HIV-infected adults were examined using multivariable logistic regression.

Demographic characteristics of the 4006 HIV-infected adults in MMP differed from the 5657 adults from the general U.S. population in NHANES, including more men (73.2% in MMP versus 49.4% in NHANES, respectively), black or African Americans (41.5% versus 11.6%), persons with annual incomes <$20,000 (64.5% versus 21.9%), and homosexuals or bisexuals (50.9% versus 3.9%). HIV-infected men were less likely to be obese (PR 0.5, CI 0.5–0.6) and HIV-infected women were more likely to be obese (PR1.2, CI 1.1–1.3) compared with men and women in the general population, respectively. Among HIV-infected women, younger age was associated with obesity (<40 versus >60 years). Among HIV-infected men, correlates of obesity included black or African American race/ethnicity, annual income >$20,000 and <$50,000, heterosexual orientation, and geometric mean CD4+ T-lymphocyte cell count >200 cells/μL.

Obesity is common, affecting 2 in 5 HIV-infected women and 1 in 5 HIV-infected men. Correlates of obesity differ for HIV-infected men and women; therefore, different strategies may be needed for the prevention and treatment.

## Abstract 2

Supplemental digital content is available in the text

## INTRODUCTION

Since the introduction of combination antiretroviral therapy (ART) in the mid-1990s, human immunodeficiency virus (HIV) associated morbidity and mortality has dramatically declined.^1,2^ Life expectancy of HIV-infected adults receiving ART has increased and is approaching that of the general population.^3–6^ As the population receiving ART ages and lives longer with HIV infection, chronic and age-related conditions are increasingly prevalent and contribute substantially to overall morbidity and mortality.^7–11^ Less than one-quarter of deaths among HIV-infected persons receiving care are due to AIDS whereas up to half are due to noninfectious causes such as cardiovascular disease (CVD), non-AIDS-related malignancies, and renal disease.^8,9^

Obesity is an independent-risk factor for CVD and obese individuals in the general U.S. population are nearly twice as likely to experience CVD, even after adjustment for other traditional-risk factors.^12^ For health care providers and patients, it is important to prevent and treat obesity, as there is an increase in risk for additional comorbid conditions and mortality in persons with body mass index (BMI) >30 kg/m^2^; for HIV-infected obese persons, this risk may be even greater than in the general population. Several estimates of the prevalence of obesity among HIV-infected adults have been reported in the literature, ranging from 9% among HIV-infected men in the military^13^ to 33% among HIV-infected women in Alabama^14^ (eTable 1, http://links.lww.com/MD/A327); however, these estimates have been derived from small studies or subpopulations with limited generalizability rather than from large, national, and population-based studies.^7,11,13–24^

Estimating the prevalence and correlates of obesity is a first step in identifying important comorbid conditions that affect HIV-infected persons and may contribute to increased mortality from non-AIDS-defining conditions. The objectives of this analysis were three-fold: to estimate the prevalence of obesity among HIV-infected individuals receiving medical care at HIV outpatient clinics using nationally representative data; to compare obesity prevalence in HIV-infected individuals receiving medical care at HIV outpatient clinics to that in the general population; and to identify factors associated with obesity in HIV-infected men and women receiving medical care at HIV outpatient clinics.

## METHODS

We describe the prevalence of obesity in a nationally representative sample of HIV-infected adults receiving care using population-based data from the 2009 data collection cycle of the Medical Monitoring Project (MMP)^25–28^ and compare it to the prevalence of obesity in a nationally representative sample of the U.S. population from the 2009 to 2010 National Health and Nutrition Examination Survey (NHANES).^29,30^

### Medical Monitoring Project (MMP)

**FIGURE 1:**
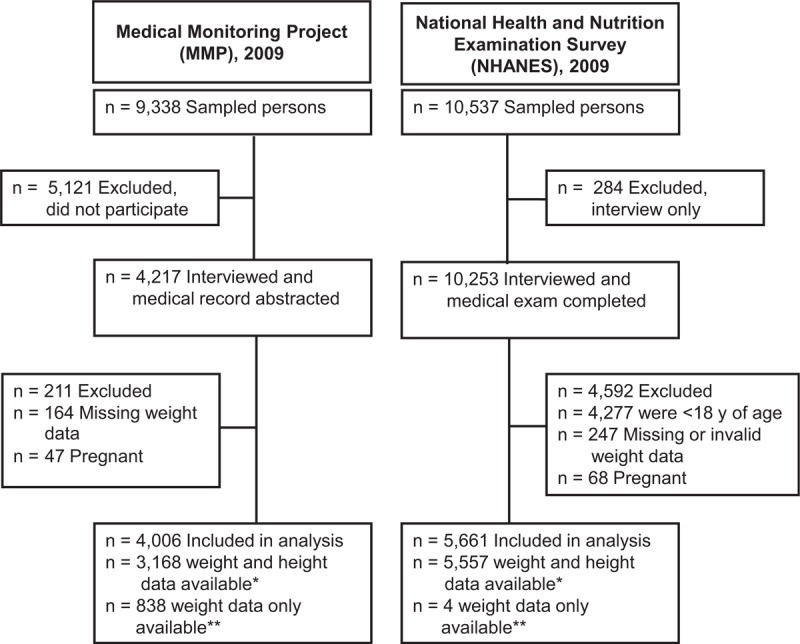
Flow diagram of participants included in this analysis of obesity among HIV-infected adults receiving medical care and adults in the general population of the United States. ^∗^Body mass index (BMI) directly calculated as weight in kilograms divided by height in meters squared (kg/m^2^). ^∗∗^BMI category inferred using available weight data and standard distribution of heights for age and sex in United States.

MMP is an HIV surveillance system designed to produce nationally representative estimates of behavioral and clinical characteristics of HIV-infected adults receiving medical care from HIV outpatient facilities in the United States.^25–28^ MMP is funded by the Centers for Disease Control and Prevention (CDC). MMP methods, including weighting procedures, have been described in detail elsewhere.^25–28^ Briefly, MMP has a cross-sectional design in which U.S. states and territories were sampled first, then eligible facilities within each state were selected, and finally, eligible individuals were selected from facilities. Eligible facilities were those that provided outpatient HIV care, defined as the treatment and management of HIV infection, including monitoring CD4+ T-lymphocyte cell (CD4) count and HIV viral load tests, or the prescription of ART. For the 2009 data collection cycle, eligible individuals were HIV-infected adults, aged 18 years or older, receiving medical care in participating facilities in 16 states and Puerto Rico, between January and April 2009. Data from MMP were weighted to produce nationally representative estimates of all HIV-infected adults receiving care in the United States. In the 2009 data collection cycle, between June 2009 and May 2010, 100% of sampled states and territories participated, 461 out of 603 sampled facilities participated (facility response rate, 76%), and 4217 of 9338 sampled persons completed an interview and had their medical records abstracted (patient-level response rate, 51%). Women who reported having been pregnant in the prior 12 months (n = 47) and persons missing weight data in their medical records (n = 164) were excluded from analysis (Figure 1). We analyzed data on 4006 participants and weighted the data to represent 399,062 HIV-infected adults in HIV medical care in the United States during January to April 2009.

Face-to-face interviews were conducted to collect information on 11 behavioral and clinical domains, characteristics including gender at birth, race, educational attainment, annual income, sexual orientation, and time since HIV diagnosis were self-reported during interviews. Data on weight, height, stage of disease, prescription of ART, and viral suppression were collected through abstraction of medical records for the year prior to the date of interview.

In accordance with the federal human subjects protection regulations at 45 Code of Federal Regulations 46.101c and 46.102d 13 and with the Guidelines for Defining Public Health Research and Public Health Non-Research,^31,32^ MMP was determined to be a non-research, public health surveillance activity used for disease control program or policy purposes. As such, MMP is not subject to review by a federal investigational review board and received approval for its protocol from CDC officials who were not involved with MMP implementation.^31^ Participating states or territories and facilities obtained local institutional review board approval to conduct MMP if required locally.

### National Health and Nutrition Examination Survey (NHANES)

NHANES, conducted by the National Center for Health Statistics, CDC, is a series of cross-sectional nationally representative health examination surveys designed to assess the health and nutritional status of the civilian, noninstitutionalized U.S. population.^29,30^ NHANES relies on a complex, multistage probability sampling design to select a representative sample, selecting counties as the primary sampling unit, then a block or group of blocks within the county, then specific households within each block, and finally individuals within each household. Weight and height were measured in a mobile examination center using standardized techniques and equipment. Participants reported demographic information during the family interview, and sexual orientation was reported by the participant using a computer-assisted personal interviewing system in the mobile examination center. From 10,537 sampled persons, 10,253 persons had both interview and body measurement data; we excluded from analysis persons who were younger than 18 years of age (n = 4277), pregnant (n = 68), or had missing or invalid weight data (n = 247, Figure 1). Of the NHANES participants included in this analysis, 85% reported having a usual place of health care. NHANES 1999 to 2010 underwent National Center for Health Statistics institutional review board/research ethics review board approval; written informed consent was obtained from the participants.

### Analytical Methods

For MMP and NHANES, distribution of sociodemographic characteristics was summarized using unweighted counts and weighted percentages with 95% confidence intervals (CIs). Factors associated with obesity and common to both surveillance systems included gender, age at the time of interview, race/ethnicity, educational attainment, and sexual orientation.^14,33–37^ We additionally examined HIV clinical care factors reported to have variable associations with obesity or weight gain in small studies or subpopulations, including years since HIV diagnosis, CD4 count, HIV disease stage per CDC criteria,^38^ ART prescription, and recent viral suppression (most recent viral load during the past 12 months was undetectable or ≤200 copies/mL).^13,14,16,17,23,39^

The weighted prevalence and 95% CI of obesity were estimated separately for men and women, because obesity reports on the general population stratify by gender^14^ and because evidence from other studies indicated that the prevalence of obesity among HIV-infected persons may differ by gender.^7,13,14,17,18,20^ BMI was calculated as weight in kilograms divided by height in meters squared (kg/m^2^). Following current recommendations, obesity was defined as a BMI ≥ 30.0 kg/m^2^.^40^ When height was missing (Figure 1), BMI category was inferred from recorded weight using the assumption that the heights of respondents fell between the 2.5th and 97.5th percentiles of age and sex-matched members of the U.S. population.^41^ Participants with missing or invalid weight measurements were excluded from analysis (eTables 2 and 3, http://links.lww.com/MD/A327).

For the characteristics common to both surveys, stratum-specific prevalence ratios (PRs) were summarized with standardized prevalence ratios (SPRs, 95% CI), utilizing the weighted prevalence and corresponding standard error (SE).^42^ This approach is an extension of the indirect method for calculating age-adjusted standardized mortality ratios, in which the observed mortality rate in a population of interest is compared with an expected one derived from the age-specific mortality rates of a standard population.^43^

Among HIV-infected men and women receiving care who participated in MMP, we assessed bivariate associations with Rao-Scott X^2^ tests to identify factors associated with obesity.^44^ Variables with *P* ≤ 0.15 in bivariate tests were included in multivariable logistic regression models examining factors independently associated with obesity in HIV-infected men and women. All two-way interactions of significant variables were considered for inclusion; tests for interaction were performed with likelihood ratio tests by comparing 2 nested multivariate models with and without the interaction term, rejecting the null hypothesis with *P* ≤ 0.05. We used predicted marginal probabilities to calculate the adjusted prevalence of obesity in multivariable logistic models.^45^ To assess the effect of missing weight data on our results, we conducted sensitivity analyses to examine differences in sociodemographic characteristics between participants who had complete weight and height data (BMI directly calculated), complete weight data only (BMI inferred), and those who were missing weight data (excluded from analysis). We then removed persons with inferred BMI from the analytic dataset and repeated analyses.

All analyses accounted for complex survey designs, weighted analyses were conducted using SAS-callable SUDAAN (RTI International, Research Triangle Park, NC), PROC SURVEYFREQ and PROC RLOGIST, in SAS 9.3 (SAS Institute, Cary, NC) for MMP and NHANES data. SPR calculations were performed in Microsoft Excel 2010 (Microsoft Corporation, Redmond, WA).

## RESULTS

Among the 4217 MMP participants with matched interview and medical record data during the 2009 data collection cycle, 4006 (95.0%) had sufficient information for calculation or inference of BMI and were included in the analytic sample (Figure 1). From 9075 persons interviewed and examined in NHANES in the 2009 to 2010 cycle, 5661 (62.4%) met inclusion criteria and had sufficient information for calculation of BMI. As shown in Table 1, the MMP population differed from the NHANES population on a number of demographic factors. Compared to NHANES, MMP included a greater proportion of males (73.2% versus 49.4%), persons aged 40 to 59 years (66.0% versus 39.4%), blacks or African Americans (41.5% versus 11.6%), persons with low income (annual income <$20,000: 64.5% versus 21.9%), and homosexual or bisexual participants (50.9% versus 3.9%). The majority of MMP participants had been diagnosed with HIV for more than 10 years (54.0%), had stage 3 (AIDS, 67.9%), were prescribed ART (83.8%), and had suppressed viral load at their most recent test (72.4%).

**TABLE 1:**
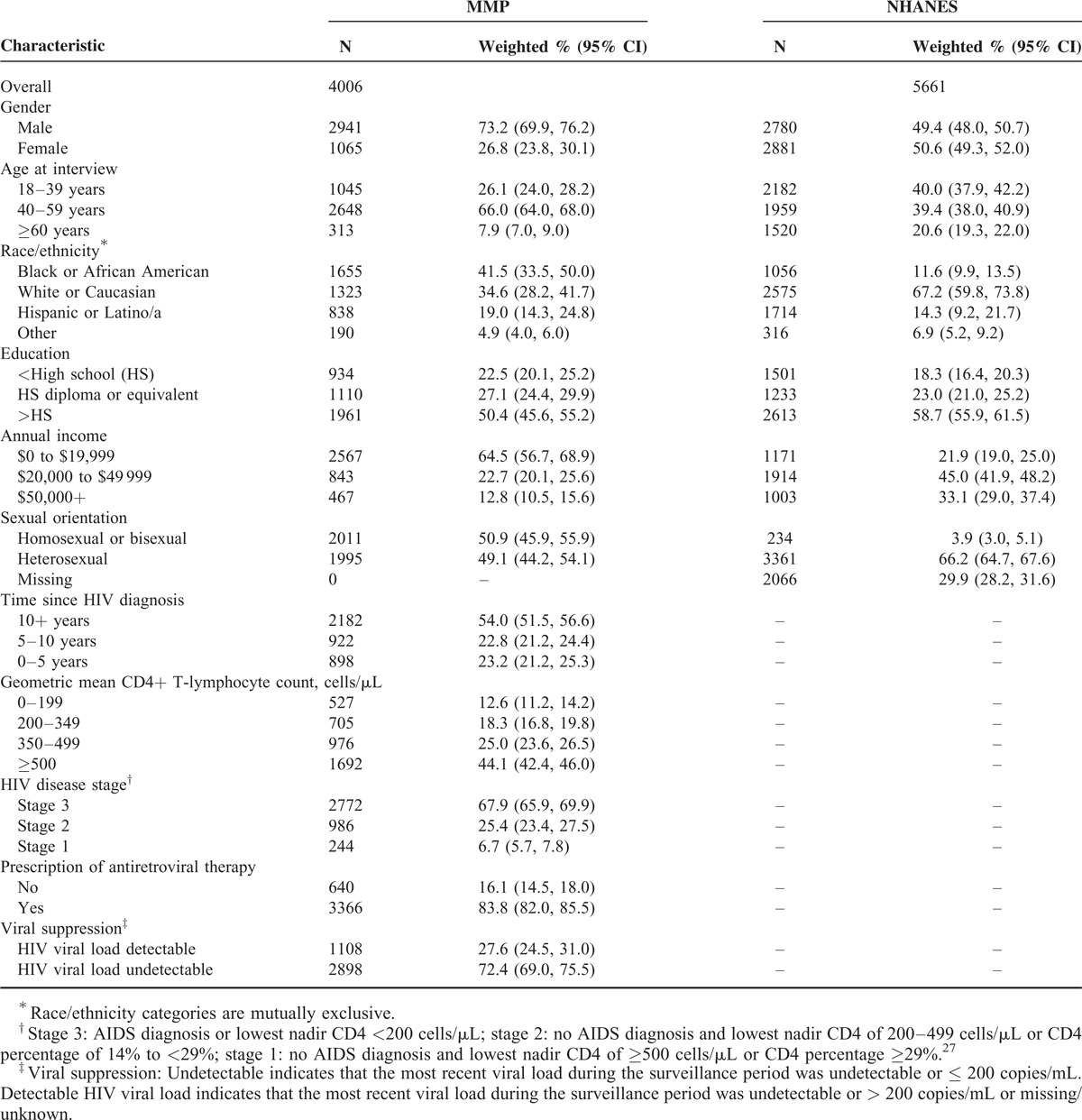
Characteristics of HIV-Infected Adults Receiving Medical Care and Adults in the General Population of the United States—Medical Monitoring Project (MMP), 2009 and National Health and Nutrition Examination Survey (NHANES), 2009

Table 2 presents the weighted prevalence (SE), stratum-specific PR, and SPR for obesity in MMP compared with NHANES for men and women. Overall, the prevalence of obesity among HIV-infected adults receiving care, compared to those in the general population was 46% lower in men (PR: 0.54, 95% CI 0.48, 0.61) and 17% higher in women (PR: 1.17, 95% CI: 1.06, 1.30). HIV-infected men receiving care were significantly less likely to be obese compared to men in the general population regardless of age, race/ethnicity, education, income level, or sexual orientation. Among women, SPR for obesity differed by subgroups. HIV-infected women receiving care were more likely to be obese than women in the general US population after standardization for age (SPR: 1.21, 95% CI 1.09, 1.34), or sexual orientation (SPR: 1.25, 95% CI 1.13, 1.40); but, less likely to be obese in prevalence estimates standardized by race/ethnicity (SPR: 0.87, 95% CI 0.78, 0.98).

**TABLE 2:**
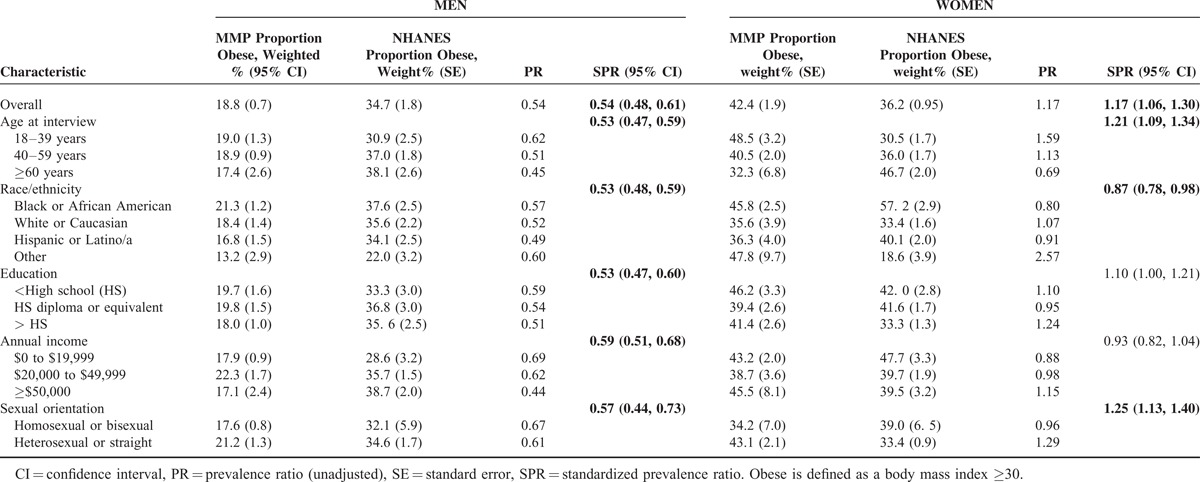
Relative and Standardized Prevalence of Obesity by Gender and 5 Demographic Characteristics – Medical Monitoring Project (MMP), 2009 and National Health and Nutrition Examination Survey (NHANES), 2009–2010

Correlates of obesity differed for HIV-infected men (Table 3) and women (Table 4). In multivariable models, race/ethnicity, income, sexual orientation, and CD4 count were significant correlates of obesity in HIV-infected men receiving medical care; those with annual income under $50,000 but over $20,000, heterosexual men, and those with CD4 count greater than 200 cells/μL were more likely to be obese. Compared to black or African American men, Hispanic, or Latino HIV-infected men receiving medical care were less likely to be obese. Among women, only age remained a significant correlate of obesity after controlling for factors significant in bivariate models; HIV-infected women receiving medical care aged less than 40 years were more likely to be obese compared to those aged 60 years or more (*P* = 0.04). We found no statistically significant two-way interactions; therefore, they were not reported.

**TABLE 3:**
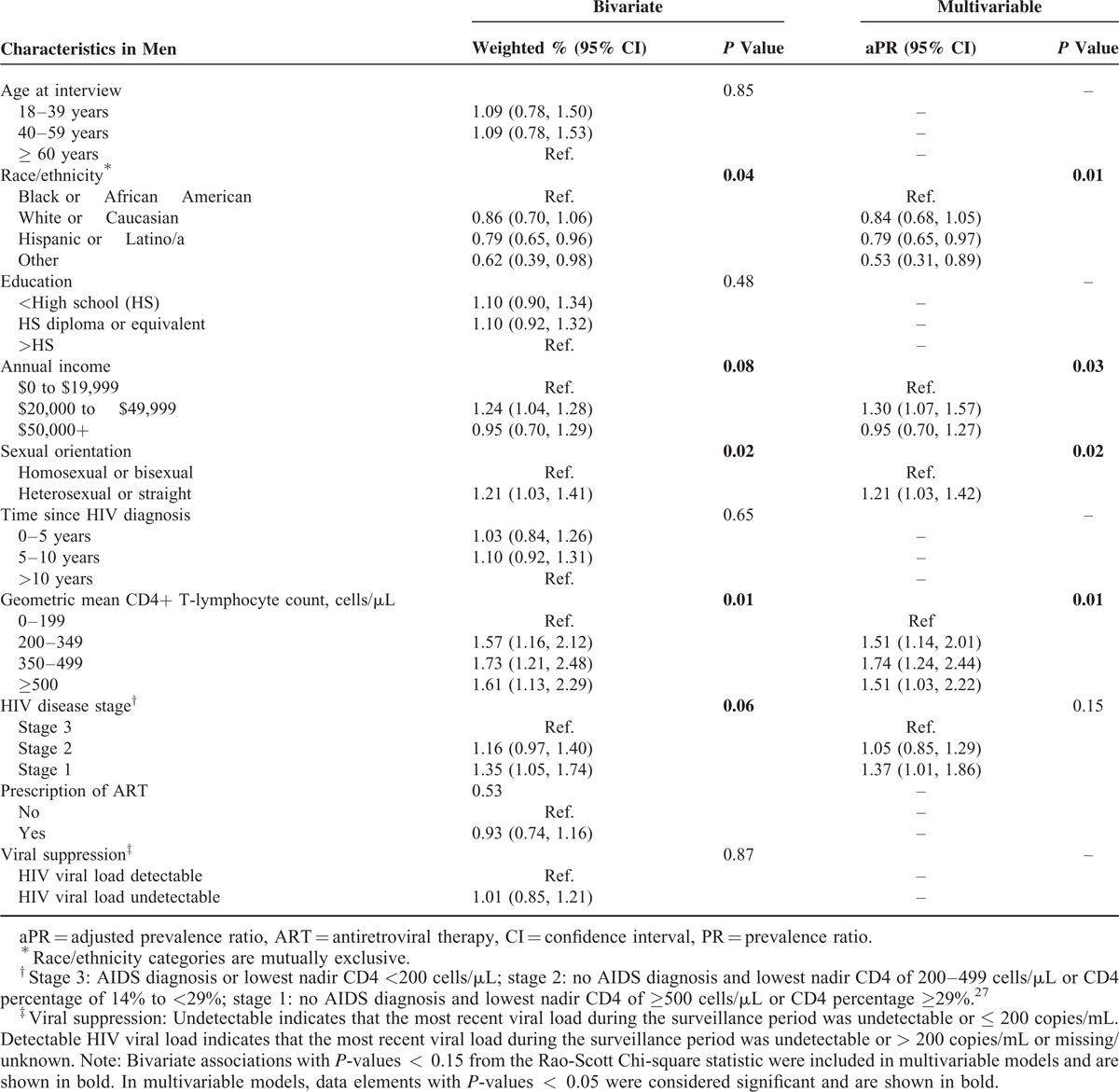
Bivariate and Multivariable Associations of Sociodemographic Characteristics and Obesity in HIV-Infected Men in Care in the United States — Medical Monitoring Project (MMP) 2009

**TABLE 4:**
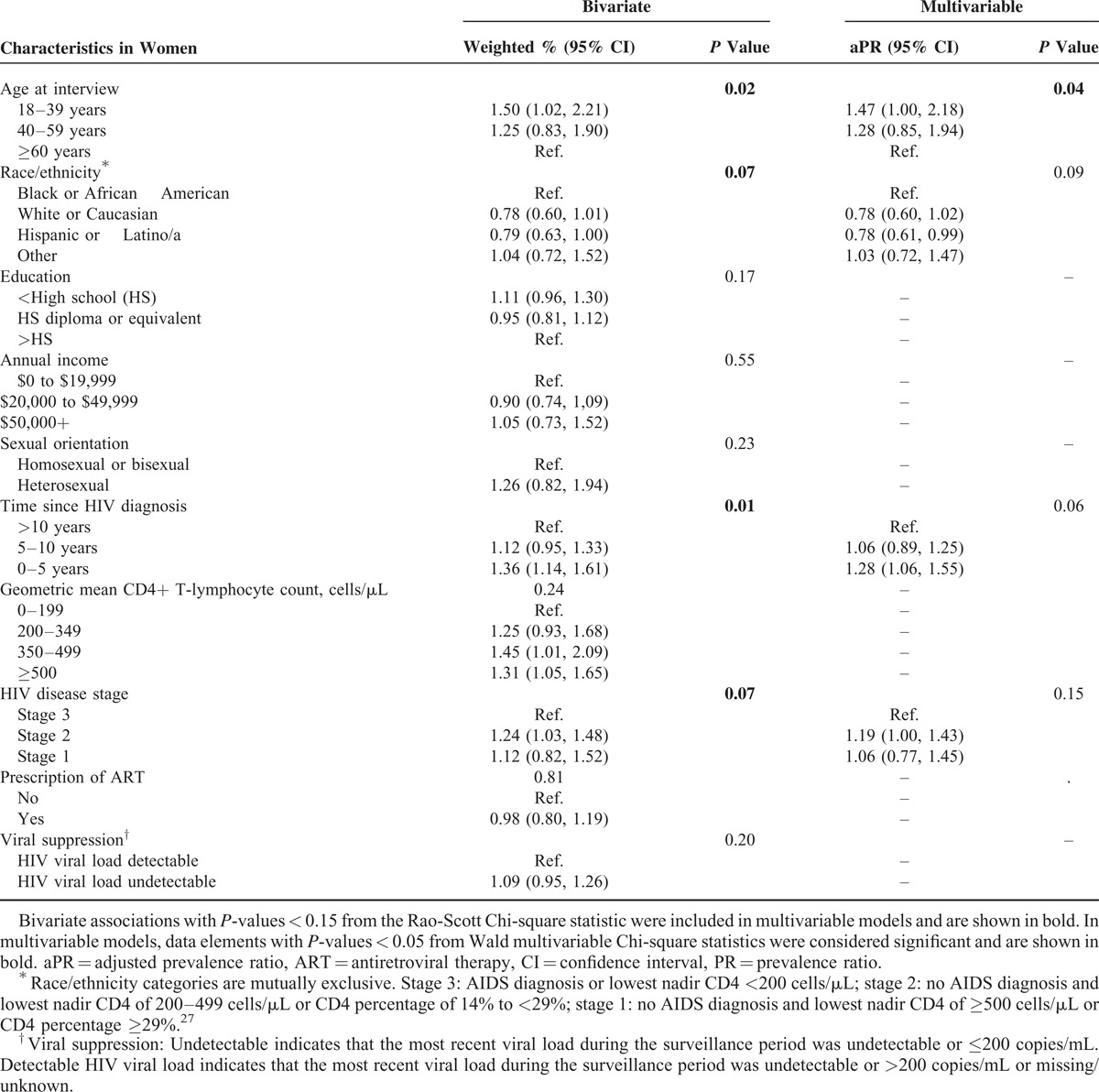
Bivariate and Multivariable Associations of Sociodemographic Characteristics and Obesity in HIV-Infected Women in Care in the United States — Medical Monitoring Project (MMP) 2009

Among MMP participants, there were no differences in sociodemographic characteristics between individuals who had complete weight and height data (BMI directly calculated) and complete weight data only (inferred BMI). Compared to persons with complete weight and height data, those who were missing weight data (excluded from analysis) included significantly more women, more persons under the age of 40 years, and a higher proportion had detectable viral loads (eTable 4, http://links.lww.com/MD/A327). Among NHANES participants, few comparisons could be made due to small numbers of persons with complete weight data only and missing data among those who were excluded from the analysis (eTable 5, http://links.lww.com/MD/A327). In sensitivity analyses, the removal of persons with inferred BMI did not result in any substantial changes in findings; therefore, these results are not presented.

## DISCUSSION

In this report, we present the first nationally representative estimates of obesity among HIV-infected adults receiving care in the United States. Similar to some previous studies,^7,19^ we found that 19% of HIV-infected men in medical care were obese and were approximately half as likely to be obese as men in the general U.S. population. However, our finding that 42% of HIV-infected women in medical care were obese is higher than previous estimates of obesity among HIV-infected women.^7,13,14,16–18,20,21^

Although obesity is nearly ubiquitous, affecting 1 in 3 adults in the general U.S. population, important differences in prevalence were observed by gender, race/ethnicity, and age, with the greatest prevalence among women, racial and ethnic minorities, and persons with low socioeconomic status.^46^ Among HIV-infected persons receiving medical care, sexual orientation, and HIV-related factors were associated with obesity. Although the prevalence of obesity was similar for men and women in the general population, HIV-infected women receiving care were twice as likely to be obese as HIV-infected men receiving care. Black or African American women in the general and HIV-infected populations have a higher prevalence of obesity than White/Caucasian or Hispanic/Latina women. Although obesity was more common among HIV-infected women, after standardization for race, HIV-infected women were less likely to be obese than women in the general population, suggesting that the distribution of race and obesity differed among HIV-infected women compared with the general population. The association between age and obesity prevalence is inverse in HIV-infected women; whereas in the general population the highest prevalence of obesity is among those 60 years and older, in HIV-infected women the highest prevalence is among those younger than 40 years. The lower prevalence of obesity among HIV-infected women aged 60 years and older may be due to a survivor bias or cohort effect.

Similar to other studies,^14,47^ we found sexual orientation to be an important correlate of obesity among HIV-infected men: heterosexual men were more likely to be obese than homosexual or bisexual men, even after controlling for sociodemographic and HIV-related factors. Contrary to studies which reported that lesbian or bisexual women were more likely to be obese,^47^ we found that HIV-infected heterosexual women were more likely to be obese than HIV-infected homosexual women or women in the general population. Although obesity prevalence differed by sexual orientation among HIV-infected women, sexual orientation was not a significant correlate of obesity among HIV-infected in this analysis, perhaps due to small sample size.

With effective treatment and control of HIV infection, HIV-infected persons can live longer and experience fewer AIDS-related complications.^3–11,48^ Obel et al^48^ recently reported that among those with no comorbidity or substance abuse, persons with optimally treated HIV infection were not at substantially increased risk of mortality compared with the general population. However, risk of myocardial infarction may be increased by 50% or more among HIV-infected persons compared with uninfected persons.^15^ Compared to HIV-infected persons with no risk factors or comorbid conditions, mortality risk may be increased by nearly 40% among HIV-infected persons with HIV-risk factors and 70% among HIV-infected persons with comorbid conditions.^48^ Non-AIDS-related comorbid conditions such as CVD, non-AIDS-related cancers, kidney disease, diabetes, hypertension, and dyslipidemia have increased among persons living with HIV since the introduction of highly active ART in the mid-1990s when the number of AIDS-related deaths began to decline.^7,9^ Although HIV- and/or ART-associated factors, such as chronic inflammation and immune activation, may contribute to increased risk for CVD^49^ among HIV-infected persons, traditional CVD-risk factors such as obesity or smoking may confer additional risk for HIV-infected persons. HIV-infected persons are twice as likely to smoke as the general population,^50^ and HIV-infected smokers may be more than twice as likely to experience a CVD event. Furthermore, HIV-infected smokers have reduced life expectancy compared with former or never smokers.^51^ Because obese individuals are at increased risk for CVD morbidity and mortality,^12^ obese HIV-infected individuals in care may be at further elevated risk due to the compounded effects of HIV infection, ART use, and increased prevalence of CVD-risk factors such as smoking.

In studies enrolling HIV-infected patients in the early to mid-1990s before ART, excess weight was associated with slower HIV progression;^16,52^ however, since the implementation of ART, that relationship may have changed. Although we found that CD4 count >200 cells/μL was associated with obesity in men and a recent cross-sectional analysis by Blashill et al^22^ found that a 1-unit increase in BMI was associated with an 8.7-unit increase in CD4 count, prospective studies have indicated that CD4 count among obese men did not significantly differ from that of normal weight men at 14 months^22^ and that obese HIV-infected persons gained significantly fewer CD4 cells compared to normal weight and overweight persons 4 years after ART initiation.^53^ This evidence does not indicate that excess weight leads to improvements in HIV infection or outcomes overtime, it may simply be that more individuals are obese at time of infection or gain weight after initiating ART.^22,53^

For HIV-infected individuals in care and with well-controlled infection, a complementary strategy to address risk factors such as obesity is needed. Guidelines for the Management of Overweight and Obesity^54^ advise calculating BMI and measuring waist circumference at least annually for patients in the general population in addition to counseling for health goals, including weight loss, physical activity, and dietary modification. In HIV-infected populations, aerobic and resistance exercise have been shown to produce positive benefits for multiple outcomes, including body composition, functional capacity, muscular strength, and cholesterol.^55^ Although the effect of exercise and physical activity on immune function has not been determined, exercise has been shown to be safe and beneficial in HIV-infected adults. The guidelines for the general population could be followed until evidence accumulates to support guidelines specific to an HIV-infected population.

This study has several limitations. First, temporal associations cannot be established since MMP is cross-sectional. Also MMP had lower than optimal response rates, but data were weighted to adjust for nonresponse. Empirical research indicates that low response rates are not necessarily indicative of nonresponse bias, particularly when probabilistic samples are drawn from rigorously constructed sampling frames.^56^ Although we were able to stratify our prevalence estimates by gender and standardize by one variable at a time, we were unable to standardize by multiple factors simultaneously, which might have aided interpretation of findings. Weight and height were abstracted from medical records across many different medical clinics in MMP and a standardized protocol for height and weight measurement was not used. Approximately 5% of MMP participants had no weight data and were excluded from the analysis. For those included in the analysis, nearly 21% were missing height data and BMI was inferred based on most likely BMI category according to recorded weight. Although some misclassification may have occurred, sensitivity analyses excluding persons with inferred BMI did not change the findings. Additionally, there were few differences in sociodemographic characteristics for participants who had complete weight and height data (BMI directly calculated), complete weight data only (inferred BMI), and those who were missing weight data (excluded from analysis). Physical activity and diet composition are associated with body composition and weight,^18,33,55,57^ but are not available in MMP; therefore, we were not able to assess their association with obesity in these analyses.

In this analysis, we examined factors traditionally associated with obesity such as age, race/ethnicity, education, and income. We also considered sexual orientation, time since HIV diagnosis, HIV disease stage, current ART use, and current viral suppression. To our knowledge, this is the only nationally representative study examining obesity prevalence in an HIV-infected population receiving care.

In conclusion, we found that, HIV-infected men receiving medical care at HIV outpatient clinics were about half as likely to be obese but HIV-infected women receiving medical care at HIV outpatient clinics may be more likely to be obese, compared respectively to men and women in the general U.S. population. It is important to note that the prevalence of obesity in HIV-infected women was more than twice that of HIV-infected men. As ART becomes increasingly effective and more easily tolerated, achieving viral suppression is only the first step to ensuring long-term health for HIV-infected patients. Correlates of obesity differ for HIV-infected men and women; therefore, different strategies may be needed for obesity prevention and treatment to reduce morbidity and mortality from adverse long-term outcomes such as CVD.
